# STAT3–mediated up-regulation of DAB2 via SRC-YAP1 signaling axis promotes *Helicobacter pylori*-driven gastric tumorigenesis

**DOI:** 10.1186/s40364-024-00577-x

**Published:** 2024-03-13

**Authors:** Yantao Duan, Pengfei Kong, Mingzhu Huang, Yonghao Yan, Yi Dou, Binhao Huang, Jing Guo, Wei Kang, Caixia Zhu, Yuyan Wang, Donglei Zhou, Qiliang Cai, Dazhi Xu

**Affiliations:** 1 Department of Gastric Surgery, Fudan University Shanghai Cancer Center, Shanghai, 200032 China; 2 Department of Oncology, Shanghai Medical College, Fudan University, Shanghai, 200032 China; 3 Department of Anatomical and Cellular Pathology, The Chinese University of Hong Kong, Hong Kong, 999077 China; 4 Key Laboratory of Medical Molecular Virology (MOE/NHC/CAMS), School of Basic Medical Sciences, Shanghai Medical College, Biosafety Level 3 Laboratory, Shanghai Institute of Infectious Disease and Biosecurity, Fudan University, Shanghai, 200032 China

**Keywords:** Gastric cancer, *H pylori*, DAB2, STAT3, YAP1

## Abstract

**Background:**

*Helicobacter pylori* (*H pylori*) infection is the primary cause of gastric cancer (GC). The role of Disabled-2 (DAB2) in GC remains largely unclear. This study aimed to investigate the role of DAB2 in *H pylori*-mediated gastric tumorigenesis.

**Methods:**

We screened various datasets of GC to analyze DAB2 expression and cell signaling pathways. DAB2 expression was assessed in human GC tissue microarrays. *H pylori* infection in vivo and in vitro models were further explored. Immunostaining, immunofluorescence, chromatin immunoprecipitation, co-immunoprecipitation, Western blot, quantitative polymerase chain reaction, and luciferase reporter assays were performed in the current study.

**Results:**

The bioinformatic analysis verified that *DAB2* was 1 of the 8 genes contributed to tumorigenesis and associated with poor prognosis in GC. The median overall survival and disease-free survival rates in DAB2^high^ group were significantly less than those in DAB2^low^ group. These findings demonstrated that *H pylori* transcriptionally activated *DAB2* expression via signal transducer and activator of transcription 3 (STAT3)-dependent pathway. By bioinformatics analysis and knockdown or overexpression of *DAB2*, we found that *DAB2* upregulated Yes-associated protein 1 (YAP1) transcriptional activity. Mechanistically, DAB2 served as a scaffold protein for integrin beta 3 (ITGB3) and SRC proto-oncogene non-receptor tyrosine kinase (SRC), facilitated the phosphorylation of SRC, promoted the small GTPase ras homolog family member A (RHOA) activation and phosphorylation of YAP1, and ultimately enhanced the YAP1 transcriptional activity.

**Conclusions:**

Altogether, these findings indicated that DAB2 is a key mediator in STAT3–regulated translation of YAP1 and plays crucial roles in *H pylori*-mediated GC development. DAB2 might serve as a novel therapeutic target for GC.

**Supplementary Information:**

The online version contains supplementary material available at 10.1186/s40364-024-00577-x.

## Introduction

With more than one million new cases and 769,000 deaths in 2020, gastric cancer (GC) ranks the fifth most prevalent malignancy and the fourth-highest mortality cancer worldwide [1]. GC ranks the sixth most prevalent cancer and the third most common cancer-related deaths in China in 2020, with an approximately 480,000 new cases and 370,000 deaths, accounting for about 50% of newly diagnosed cases and the cancer-related deaths worldwide in 2020 [2]. The prognosis of GC patients was still unsatisfactory, with a 5-year survival rate of lower than 30% [3]. The mechanisms of GC tumorigenesis remain poorly elucidated. Therefore, there is an urgent need to reveal the molecular mechanisms contributing to GC development and explore efective therapeutic treatment to improve GC clinical outcomes.

Chronic infection with *H pylori*, a class I carcinogen, is one of the most important risk factors for GC [4]. *H pylori* has infected half of the world population, and this chronic infection of gastric mucosa leads to the Correa’s cascade through multi-step pathological processes from atrophic gastritis, intestinal metaplasia, dysplasia, and eventually to GC [5, 6]. Accumulating research shows that *H pylori* infection can driver a series of oncogenic signaling pathways, including Wnt/β-catenin, Nuclear factor κB (NF-κB), c-Jun NH2-terminal kinase (JNK), and mitogen-activated protein kinase (MAPK) [7–9]. These abnormal host cellular pathways further regulate the GC development and progression.

During *H pylori* infection, inflammation has been shown to play important roles in tumour initiation and malignant transformation of GC [10]. Signal transducer and activator of transcription 3 (STAT3) is one of the most crucial molecules activated during gastric carcinogenesis, driving chronic inflammation to cancer [11, 12]. The Hippo signaling pathway dysregulation is also a common event in GC, and the Yes–associated protein 1 (YAP1) is the crucial terminal effector to regulate the organ size and cell fate [13]. Moreover, accumulating evidence shows a close linking between *H pylori* infection and the Hippo–YAP1 pathway activation [14–16]. These dysregulated signaling pathways in tumorigenesis interact with each other, rather than exist in isolation [17, 18]. Intriguingly, it has been reported that the coactivation of STAT3 and YAP1 cooperates to promote liver regeneration [19]. In human colorectal cancer, STAT3 is required for YAP1 activation, and they cooperate to regulate tumor angiogenesis [20]. Nevertheless, the molecular mechanism of their interaction in gastric tumorigenesis of *H pylori* infection remains unknown.

Disabled homolog 2 (*DAB2*) was firstly reported low expressed in ovarian cancer in 1994 [21]. The whole genome of this gene was firstly identified in 1996 [22]. *DAB2* locates on chromosome 5p13 and encodes a 96 kDa phosphoprotein with mitogenic reactivity [23]. DAB2 functions as a tumor suppressor via its inhibition on the oncogenic signaling pathways including Wnt/β-catenin and transforming growth factor beta (TGFβ) pathways [24]. However, there is contradictory findings demonstrating a pro-tumorigenic role of DAB2. DAB2 plays key roles in TGFβ-induced epithelial to mesenchymal transition (EMT), which in turn promoted the activation of focal adhesion kinase (FAK) to enhance cell survival [25]. DAB2 is highly expressed in tumor-infiltrating tumor-associated macrophages (TAMs) and its downregulation significantly inhibits lung metastasis by regulating integrin recycling and extracellular matrix (ECM) remodeling [26]. In the current study, we uncover novel oncogenic functions of DAB2 that contribute to *H pylori* infection-driven GC. These results reveal a previously unreported mechanism by which *H pylori* infection upregulates DAB2 expression to promote gastric carcinogenesis. Furthermore, we demonstrate that DAB2 induce the activation of YAP1 via binding to SRC proto-oncogene non-receptor tyrosine kinase (SRC) and integrin beta 3 (ITGB3), thus promoting ras homolog family member A (RHOA) activation and phosphorylation of YAP1, and finally promoted the YAP1 transcriptional activity. Our results suggest DAB2 may serve as a potential therapeutic target in GC.

## Materials and methods

### Cell Culture and Reagents

AGS, HGC27, MKN45, and SNU719 cells were purchased from the Shanghai Institutes for Life Sciences, Chinese Academy of Sciences (Shanghai, China). Cells were grown at 37 °C with 5% CO_2_ and maintained in RPMI/1640 cell culture medium (Gibco, Shanghai, China) supplemented with 10% Fetal Bovine Serum Premium (GIBCO, Brazil), and 100 U/ml penicillin and streptomycin (GIBCO, Shanghai, China). The reagents were purchased as follows: dasatinib (S1021) (Selleckchem, Houston, USA) and recombinant human IL-6 (Beyotime Biotechnology, Shanghai, China).

### *H pylori* Strains

*H pylori* strains 26,695, 43,504, and PMSS1 were adopted in the current study. Briefly, *H pylori* strains were cultured in Columbia agar (OXOID, Thermo Fisher Scientific, USA) medium containing 8% sterile defibrinated sheep blood under microaerophilic conditions (5% O_2_; 15% CO_2_; 80% N_2_) at 37 °C for 3 days. *H pylori* 26,695 and 43,504 strains were used for co-culture with GC cells at a multiplicity of infection (MOI) of 100:1. Furthermore, PMSS1 of 1×10^9^ colony-forming units (CFU) were used to infect every wild-type C57BL/6 mouse (Institute of Zoology, Chinese Academy of Sciences, Shanghai, China) by oral gavage.

### Human Tissue Samples

All GC tissues were obtained from Fudan University Shanghai Cancer Center (FUSCC, Shanghai, China) from January 2012 to October 2021. All experimental procedures involving human specimens were approved by the institutional review committee. Informed consent was obtained from all human participants with the permission of the Institutional Review Board of FUSCC. The study was conducted in accordance with the Declaration of Helsinki. This study included 1 tissue microarray (TMA) with 159 GC tumor tissues and 1 TMA with 77 GC of tumor and adjacent non-tumor tissues. Meanwhile, we collected 5 cases of atrophic gastritis, 5 cases of intestinal metaplasia, and 6 cases of intraepithelial neoplasia.

### Bioinformatics Analysis

All array data (GSE40634, GSE62254, GSE27342, GSE54129, GSE60427) are available at Gene Expression Omnibus (GEO) Datasets (https://www.ncbi.nlm.nih.gov/gds/). We also analyze the expression of *DAB2* in The Cancer Genome Atlas (TCGA)-GC dataset from the National Cancer Institute (https://portal.gdc.cancer.gov/). Differentially expressed genes were determined using the above GC datasets. TCGA-GC RNASeq data sets were downloaded from TCGA (http://cancergenome.nih.gov/). To analyze the prognostic value of *DAB2* and the combination of *DAB2* with YAP1 target gene cellular communication network factor 2 (*CCN2* or *CTGF*), cellular communication network factor 1 (*CCN1* or *CYR61*), and AXL receptor tyrosine kinase (*AXL*), the correlations were analyzed using the Kaplan–Meier Plotter (http://kmplot.com/analysis/). Correlations between different genes were analyzed using the TIMER website (https://cistrome.shinyapps.io/timer/).

### Immunohistochemistry Staining

Immunohistochemistry (IHC) analysis was performed using a DAB substrate kit (Dako, Denmark). Paraffin sections (4 μm) were baked for 1 h at 65 °C. After deparaffinization and rehydration, antigen retrieval was conducted by boiling in citrate buffer for 10 min. After inhibition of endogenous peroxidase activity with 3% hydrogen peroxide, the sections were blocked with 5% bovine serum albumin and incubated with primary antibodies at 4 °C overnight. The bound primary antibodies were visualized by standard avidin-biotinylated peroxidase system. The nuclei were counterstained with hematoxylin. Then, morphologic images were acquired with Olympus BX51 microscope. The antibodies used for IHC are listed in Additional file 1: Table S1.

The scoring criteria for IHC were described previously [27]. 5 fields were randomly selected to evaluate the intensity and percentage of positive cells in each slide. The intensity of staining was defined as 0 (no staining), 1 (weak staining, light yellow), 2 (medium staining, yellow to brown), or 3 (strong staining, brown), and the extent of stained cells was classified as 0 (< 5% positive cells), 1 (6–25% positive cells), 2 (26–50% positive cells), 3 (51–75% positive cells), or 4 (> 75% positive cells). The final score was determined by multiplying the intensity score with the extent of score of stained cells (from 0 to 12 totally).

## Immunofluorescence Assay

Immunofluorescence (IF) staining was performed as described previously [28]. Briefly, fixed and permeabilized gastric organoids or gastric mucosa tissue slides were permeabilized with 0.5% Triton X-100, and then blocked with 5% BSA. The primary antibodies were incubated overnight at 4 °C, followed by incubation with appropriate fluorescent dye–labeled secondary antibodies at room temperature for 2 hours. The nuclei were stained with 4, 6-diamidino-2-phenylindole (DAPI) (Thermo Fisher), and the stained cells were imaged with a Leica TCS Inverted Fluorescence Microscope. For statistical analysis, the number of positive cells was counted at 40× magnification in 5 random fields. The antibodies used for immunofluorescent staining are listed in Additional file 1: Table S1.

### Western Blotting

The cells were lysed in RIPA lysis buffer (Shanghai, China) supplemented with a protease inhibitor and phosphatase inhibitor cocktail (Pierce, Appleton, WI, USA). Protein concentrations were detected by BCA protein assay. 30 μg protein per well was separated in 10% sodium salt-polyacrylamide gel electrophoresis and transferred to PVDF membranes, which were closed with 5% non-fat milk solution (TBST dilution) for 1 h at room temperature and then incubated with primary antibodies at 4 °C overnight. Membranes were probed with specific antibodies and appropriate IRDye-800CW-conjugated secondary antibodies, and scanned with an Odyssey Infrared scanner (Li-Cor Biosciences). GAPDH was used as the loading control. Finally, the relative density of the bands was quantified using ImageJ software. The antibodies used for Western blot are listed in Additional file 1: Table S1.

### Immunoprecipitation

Cell lysates were collected in the lysis buffer (50 mM Tris–HCl pH 7.4, 5 mM EDTA, 1% TriTonX-100, 150 mM NaCl, 1x Protease inhibitors) for 1 h at 4 °C. Appropriate cell lysates were incubated with protein agarose A/G beads (Santa Cruz Biotechnology, Dallas, Texas, USA) bound with the antibody at 4 °C with slow shaking overnight. After washing 5 times with PBS, the precipitated proteins were eluted from the beads by boiling with SDS loading buffer. The eluted samples were visualized by Western blotting.

### Real-time PCR analysis

Total RNA was extracted from cells using TRIzol (Invitrogen), processed for cDNA synthesis using the Reverse Transcription Kit (Applied Biosystems), and subjected to the qRT-PCR using SYBR Green Master Mix (Applied Biosystems). According to manufacturer’s protocol, PCR conditions were as follows: 1 cycle at 95 °C for 5 min, followed by 40 cycles of 95 °C for 15 s (denaturation), 60 °C for 30 s (annealing) and 72 °C for 30 s (extension). The sequences of primers used were listed in Additional file 1: Table S2. Each sample was measured in triplicate biological replicates. Each experiment was repeated three times and the representative results were shown. To determine relative gene expression levels, the CT values were normalized with the CT values of housekeeping gene *GAPDH* using the ΔCT method.

### Plasmid Construction and Cell Transfection

The over-expressions of *STAT3* and *DAB2* were synthesized and integrated into pcDNA3.1 by Genechem (Shanghai, China), and then transfected into 293 T cell line using Lipofectamine 3000 (Invitrogen, Carlsbad, CA) following the manufacturer’s instructions. After culturing for 48 h, the transfected cells were extracted and examined by Western blot to analyze the over-expressed efficiency. Then the cells were used for further study.

### Chromatin Immunoprecipitation

The ChIP express Enzymatic Kit (Beyotime Biotechnology, Shanghai, China) was used according to our previous protocol [27]. AGS and HGC27 cells were treated with *H pylori* 26,695/43504 strains for 6 hours, STAT3/vector plasmids for 48 hours, and IL6 (100 ng/mL)/PBS for 6 h. 1% formaldehyde was used to crosslink the proteins with DNA for 10 min at room temperature. Then, DNA of the cells was sonicated and sheared to fragments of 200–1000 bp. STAT3 and normal IgG antibodies were added to the supernatant with protein G magnetic beads on a rocker at 4 °C overnight. The antibody-bound protein/DNA complex was eluted, and the DNA was purified with a PCR purification kit (TaKaRa, Beijing, China). qRT-PCR was performed to quantify binding of STAT3 to the promoter of *DAB2*. Additional file 1: Table S2 shows the primers’ sequences.

### Luciferase Reporter Assay

Luciferase reporter assays were performed as we previously described [29]. The luciferase reporter plasmids were transfected into GC cells using Lipofectamine 3000 (Invitrogen). Following the manufacturer’s instructions, luciferase activity was detected 48 h post-transfection using the Dual-Glo Luciferase Assay System (Promega, Madison, WI, USA). Firefy luciferase activity was normalized to Renilla luciferase activity for each sample. The experiments were performed in triplicate.

### Tissue Dissociation and Organoid Culture

Organoid culture was performed as previously described [30]. Briefly, 3 fresh tumor tissues from the stomach were cut into 3-mm pieces and digested with 1 mg/ml collagenase IV (Sigma, USA) for 30 min at 37 °C. The final supernatant was passed through a 70-μm filter and crypts fraction was centrifuged at 300 g for 5 min followed by resuspended with 50% Matrigel/organoid culture media (OmaStem,Guangzhou, China). 70 μL matrigel resuspension mix were dripped in the center of a 24-well plate and incubated at 37 °C and 5% CO_2_ atmosphere for 30 min. Then, 400 μL of the organoid culture media was added to each well, and the medium was changed every 2 days. Organoids from the third passage were infected with control or AAV-sh*DAB2* lentivirus (ObiO technology, Shanghai, China) with a MOI (v.g/cell) of 1.0 × 10^5^. After 7 days of incubation, the diameter of organoids in 3 random 100 magnification fields were detected using an inverted microscope. Detailed clinical information of GC patients for organoid model establishment were listed in Additional file 1: Table S3.

### Adeno-Associated Virus (AAV) -Mediated *DAB2* Knockdown

For the patient-derived primary tumor transplantation, fresh human GC tissues were obtained from GC patients who had received radical surgery R0 resection at FUSCC. Samples were collected from patients who had never received any previous treatment before operation. The use of these specimens and patients’ information was approved by the Ethics Committee of the FUSCC. Fresh GC tissues were cut into pieces (about 50 mm^3^) and subcutaneously transplanted into the right flank of severely immune-deficient NSG mice (male, 5-week-old, QIZHEN LAB, Zhengjiang, China). Detailed clinical information of GC patients for PDX model establishment were listed in Additional file 1: Table S4.

Furthermore, we used adeno-associated virus (AAV) for the knockdown of *DAB2* expression. The pAAV-U6-shRNA (*DAB2*)-CMV-WPRE (AAV-sh*DAB2*) was synthesized by Obio Technology Co. (Shanghai, China). To evaluate the effects of AAV-sh*DAB2* in vivo, PDX models were used. Mice for each patient were randomized into three groups (*n* = 5 for each patient, 2 patients): (A) normal saline control group, (B) AAV-null infection group, (C) AAV-sh*DAB2* infection group. 200 μl AAVs diluted in normal saline solution were administered to each mouse through tail vein with a single dose of 1.0×10^11^ v.g. of AAV-sh*DAB2* twice a week for three weeks. Tumor growth was monitored every week by cliper for four weeks using formula Volume = (width^2^ × length) × 0.5.

### Mouse Xenografts

We next explored the effects of *DAB2* on tumorigenesis in vivo. For limiting dilution assay, 10^3^, 10^4^, 10^5^, and 10^6^ HGC27 *DAB2*-knockdown or control cells cells were injected subcutaneously into the right flank of 4-week-old BALB/c nude mice (male, 6 mice per group). Tumor xenografts were detected once a week for 4 weeks. Tumor volume was calculated following the formula: Volume = (width^2^ × length) × 0.5. The frequency of tumor initiating cells was calculated using the Extreme Limiting Dilution Analysis (http://bioinf.wehi.edu.au/software/elda/).

### Statistical Analysis

Each experiment was performed at least three times, and the data were expressed as mean ± SD. The association between different groups and the prognosis of gastric cancer patients was evaluated using the Kaplan–Meier method. Comparisons between 2 groups were performed using unpaired Student t test or one-way analysis of variance. Correlations between groups were determined by the Pearson’s correlation test. Analyses were performed using the R statistical package (R version 3.6.1) or GraphPad Prism 6.0 (GraphPad Software, Inc., La Jolla, CA). A two-tailed *P* value < 0.05 was considered statistically significant.

## Results

### Integrated Transcriptomics Analysis Identified *DAB2* As an Oncogene in *H pylori* Infection-Driven Gastric Carcinogenesis

**Fig. 1:**
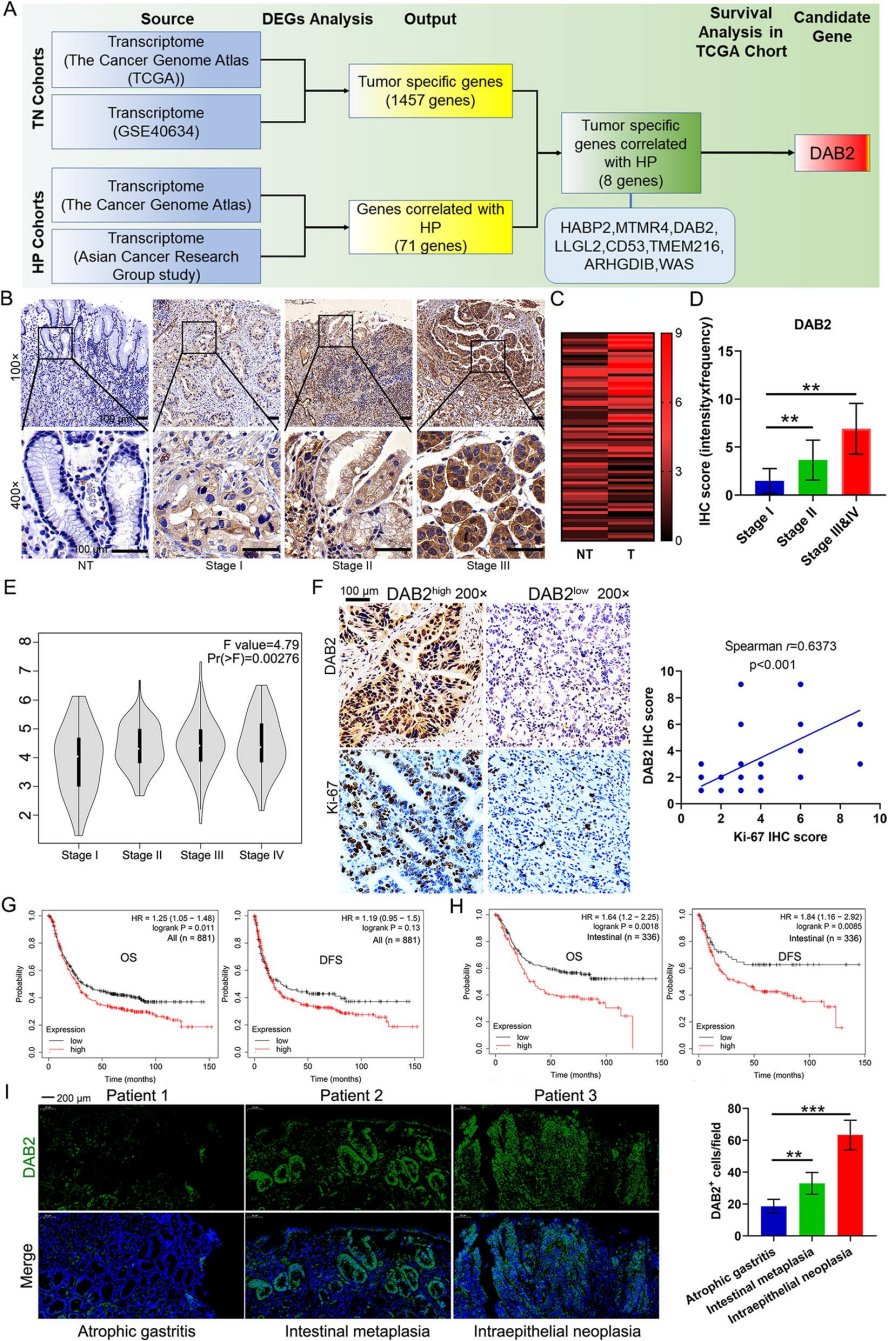
Expression and clinical significance of *DAB2* in GC. **A** Schematic illustration of strategies for screening the key genes involved in *H pylori* infection-driven gastric tumorigenesis. Gene Expression Omnibus and TCGA datasets were used to determine the upregulated genes in GC tissues (T) compared with nontumor (NT) tissues. TCGA and ACRG cohorts were uesd to determine the upregulated genes that correlated with *H pylori* infection (HP cohort). **B** Immunohistochemistry (IHC) analysis shows the expression of *DAB2* in normal and human gastric cancer tissue samples (upper panels: magnification ×100; lower panels: magnification ×400). **C** Quantification scores of DAB2 in GC are shown. **D-E** The expression levels of *DAB2* in different stages of GC in our cohort (**P < 0.01) (**D**) and TCGA (**E**) database. **F** GC tissues were stained with DAB2 and Ki-67 by IHC. Spearman’s rank correlation analysis was used to evaluate the correlation between *DAB2* and *Ki67* expression (right). Scale bar, 100 μm. **G-H** Kaplan-Meier survival curve demonstrates an association between high expression of *DAB2* and poor overall survival (OS) and disease-free survival (DFS) in overall GC (**G**) patients and intestinal-type GC (**H**). HR, hazard ratio. **I** IF analysis shows the expression of DAB2 in patients with atrophic gastritis, intestinal metaplasia, and dysplasia (magnification ×200). Quantification scores are shown on the right panel

To identify the potential driver gene in *H pylori* infection-induced gastric carcinogenesis, we performed a series of screening process using the public databases. The genes overexpressed in GC and negatively correlated with prognosis were regarded as the candidate oncogenes (TCGA and GSE40634; TN group). Meanwhile, we focused on genes that correlated with *H pylori* infection in human samples (TCGA and ACRG cohorts; HP group). We obtained 1457 and 71 differential expression genes (DEGs) from TN group and HP group, respectively. Then, we identified 8 DEGs by overlapping the two groups. To validate the clinical significance of these 8 DEGs in GC, we next explored survival analysis in TCGA cohort. As shown, we ultimately identified *DAB2* (Fig. 1A and Additional file 2: Fig. S1) as the oncogenic driver in *H pylori*-related gastric carcinogenesis, and subsequently elucidated the roles of *DAB2* in GC.

To validate the function of DAB2 in GC, we conducted an IHC analysis using a TAM from 77 pairs of GC and the adjacent normal tissues. DAB2 was overexpressed in GC tumor tissues (Fig. 1B-C). Consistently, *DAB2* expression was substantially higher in tumor tissues from GEO datasets (GSE27342 and GSE54129) and our cohort (Additional file 2: Fig. S2A-C). In addition, DAB2 expression increased with higher clinical stage both in our cohort and TCGA cohort (Fig. 1D-E). These results demonstrated that high *DAB2* expression indicates more advanced GC, suggesting that *DAB2* may play a role in tumor progression. To identify the the correlation between DAB2 expression and cancer expansion, we explored the correlation between DAB2 level and Ki-67 status in human GC TAM array and observed a positive correlation between DAB2 and Ki-67 expression, highlighting the clinical relevance of high DAB2 expression and GC cell proliferation (Fig. 1F).

To define the clinical values of *DAB2* in GC, we analyzed the correlation between *DAB2* expression and GC patients’ survival using the Kaplan–Meier Plotter. The findings demonstrated that elevated *DAB2* level was correlated with shorter overall survival (OS), but not disease-free survival (DFS) (Fig. 1G). Studies indicate that intestinal type GC is strongly linked to *H pylori* infection [31, 32]. Furthermore, we analyzed the correlation between *DAB2* and intestinal type GC survival according to Lauren’s classification. Our findings identified higher *DAB2* expression was more significantly associated with shorter OS and DFS in patients with intestinal-type GC (Fig. 1H). In this study, we also analysed the *DAB2* expression in *H pylori*-positive atrophic gastritis, intestinal metaplasia, and dysplasia in the multi-step pathological processes of Correa’s cascade, and found that *DAB2* expression increased with disease progression (Fig. 1I). These results strongly demonstrate that DAB2 is significantly upregulated in human GC, and is involved in *H pylori*-driven gastric carcinogenesis.

### *H pylori* Infection Promotes DAB2 Expression and Signal Transducer and Activator of Transcription 3 Activation

**Fig. 2:**
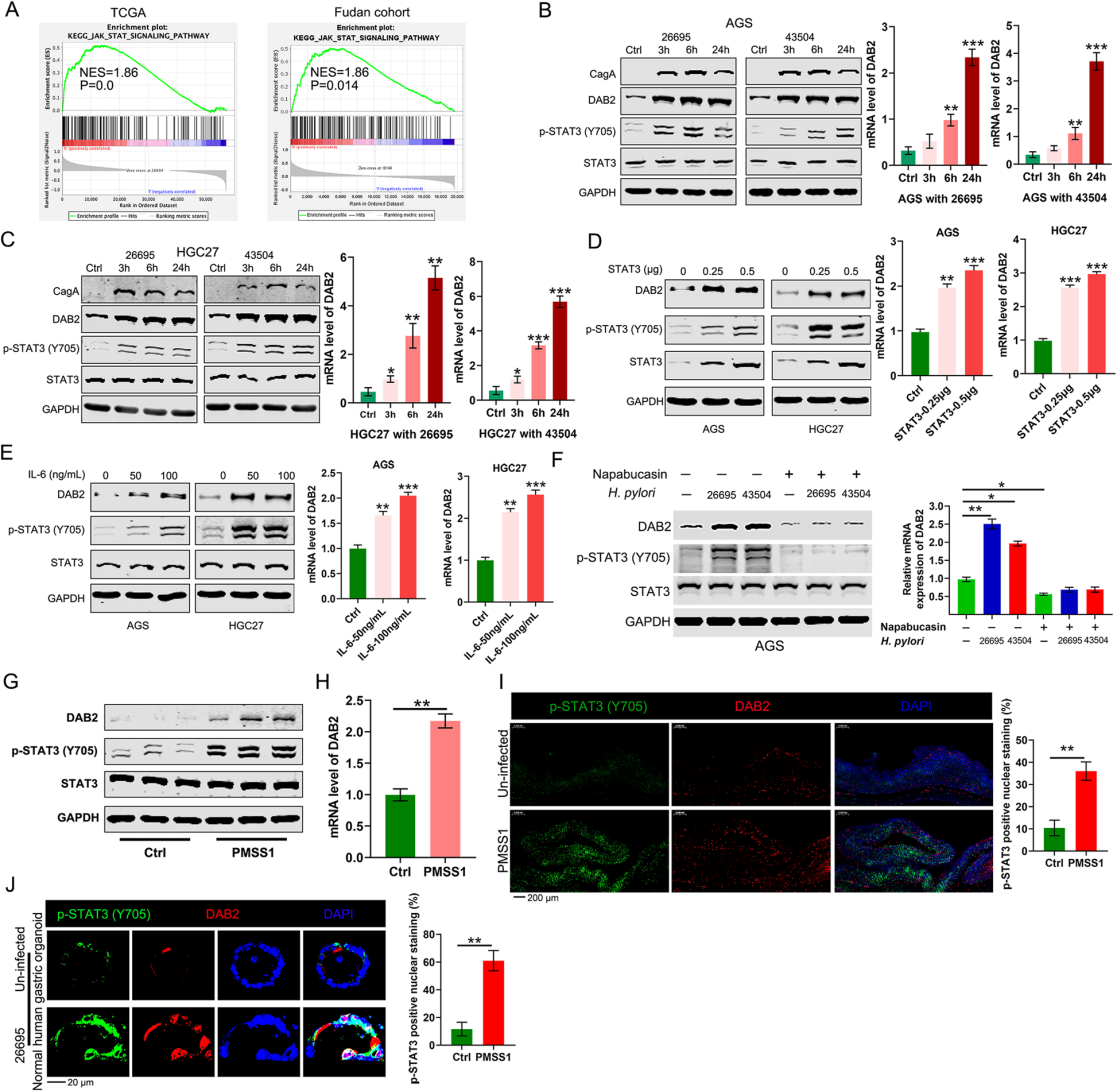
*H pylori* infection upregulated *DAB2* expression through STAT3. **A** GSEA based on gene expression analysis of GC in TCGA and Fudan database indicated that *DAB2* has a significant correlation with JAK/STAT signaling in GC. NES, normalized enrichment score. **B-C** Western blot and qRT-PCR analysis of *DAB2* in AGS and HGC27 cells following *H pylori* infection; *P < 0.05, **P < 0.01, ***P < 0.001. **D-E** Western blot and qRT-PCR analysis of DAB2 in AGS and HGC27 cells treated with STAT3 and IL6; **P < 0.01, ***P < 0.001. **F** Western blot and qRT-PCR data shows that DAB2 and p-STAT3 after *H pylori* infection and napabucasin treatment in AGS cells; *P < 0.05, **P < 0.01. **G** Western blot analysis shows DAB2 and p-STAT3 in gastric tissues of wildtype mice infected with PMSS1 *H pylori* for 2 weeks compared with noninfected mice. **H** qRT-PCR analysis of *DAB2* in gastric tissues of wildtype mice infected with PMSS1 *H pylori* for 2 weeks. **P < 0.01. **I** IF analysis shows DAB2 and p-STAT3 in gastric tissues of mice infected with PMSS1 *H pylori* for 2 weeks compared with noninfected mice (magnification ×50, scale bar, 200 μm). Quantification of p-STAT3 is shown on the right panel; **P < 0.01. **J** IF data of DAB2 and p-STAT3 staining in uninfected and *H pylori* 26,695–infected organoids derived from normal human gastric tissues. Quantification of positive p-STAT3 cells was performed (magnification ×200, scale bar, 20 μm). Quantification of positive p-STAT3 cells was shown; **P < 0.01 (right panel)

Our results indicated that *DAB2* expression was higher in 24 *H pylori*-positive gastritis samples than that in 8 *H pylori*-negative normal samples from the GSE60427 database (Additional file 2: Fig. S3). Gene set enrichment analysis (GSEA) indicated that JAK-STAT signaling was enriched in *DAB2*-high expression tissues both in TCGA and our local cohort (Fig. 2A). *H pylori* induced pro-carcinogenic STAT3 signalling activation have been shown to contribute to *H pylori*-associated pro-inflammatory and gastric carcinogenesis [33, 34]. We next asked whether *H pylori* infection regulates *DAB2* expression by activating STAT3 in AGS and HGC27 cells infected with *H pylori* (26,695 and 43,504 strains). As expected, western blot assay demonstrated that *H pylori* infection substantially promoted DAB2 protein levels in GC cells (Fig. 2B-C). Furthermore, qRT-PCR findings validated that *H pylori* infection enhanced the *DAB2* mRNA expression (Fig. 2B-C).

Next, we investigated whether *H pylori*-induced *DAB2* expression is dependent on the activation of STAT3 signaling. Western blot analysis and qRT-PCR results demonstrated that transient overexpression of STAT3 induced upregulation of *DAB2* in AGS and HGC27 cells (Fig. 2D). Consistantly, activation of STAT3 by IL-6 significantly promoted DAB2 protein and mRNA levels in AGS and HGC27 cells (Fig. 2E). As a mechanistic validation, we treated AGS cells with STAT3 inhibitor-napabucasin, and found that napabucasin inhibited p-STAT3 (Y705) levels and eliminated the *H pylori*-induced increase in DAB2 protein and mRNA levels (Fig. 2F). Furthermore, we also confirmed that *H pylori* promoted *DAB2* expression and activation of STAT3 signaling after infection of mice with *H pylori* PMSS1 (Fig. 2G-I). In addition, the IF staining illustrated a significant increase in nuclear localization of p-STAT3 and a marked improvement of *DAB2* expression after *H pylori* infection in human organoids (Fig. 2J). Taken together, these findings demonstrated that *H pylori* infection upregulated the expression of *DAB2* with the activation of STAT3 in GC cell.

### *DAB2* Expression Is Promoted via Transcriptional Activation of Signal Transducer and Activator of Transcription 3

**Fig. 3:**
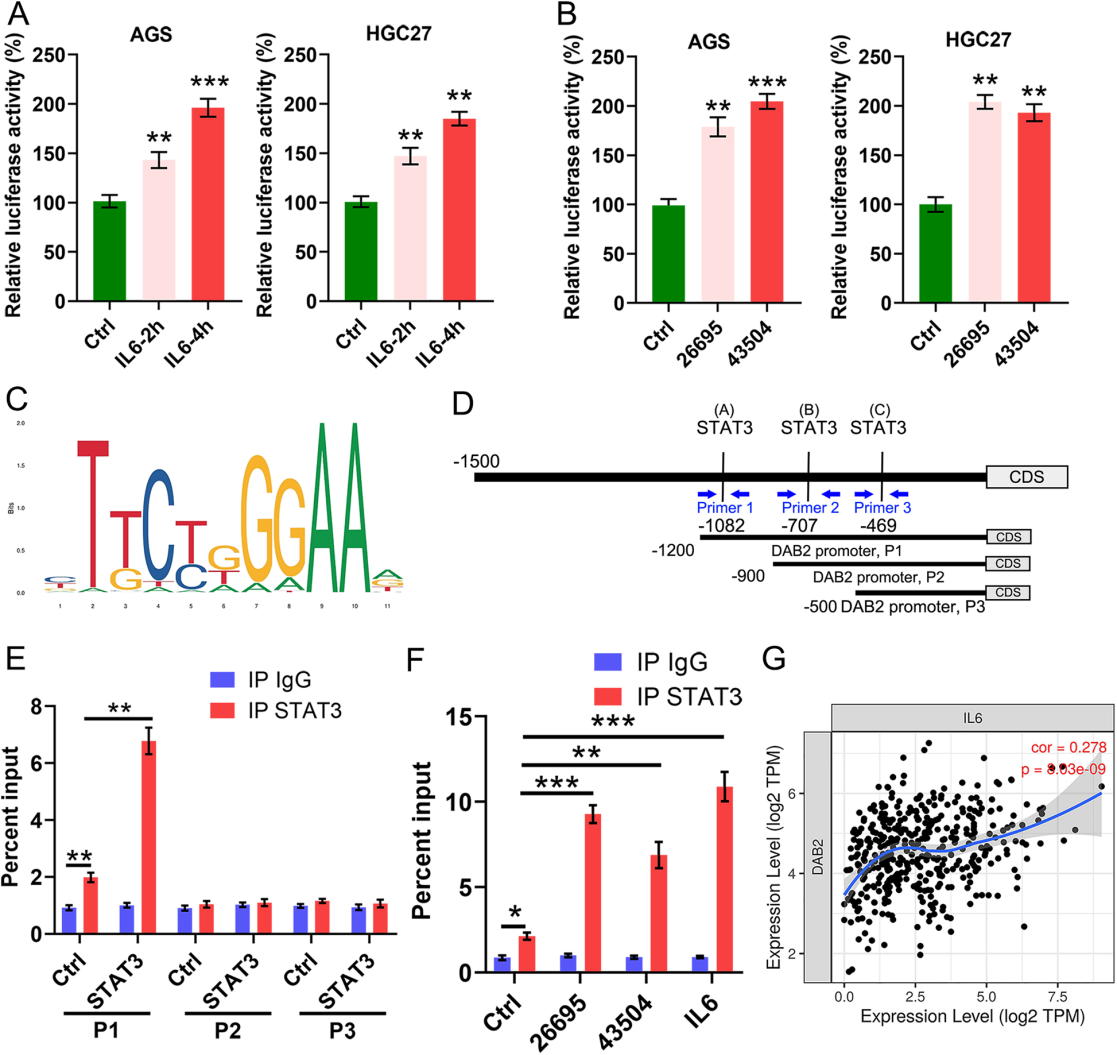
*DAB2* expression is induced via direct transcriptional activation of STAT3 on the *DAB2* promoter. **A-B** *DAB2* promoter luciferase reporter assays were performed in AGS and HGC27 cells with IL6 treatment (**A**) or *H pylori* infection (26,695 and 43,504, **B**); **P < 0.01, ***P < 0.001. **C-D** A schematic diagram shows the location of STAT3 putative binding regions on *DAB2* promoter. ChIP assay primers were designed to cover regions P1 to P3. **E** ChIP assay showing amplification of DNA fragments quantified by qRT-PCR and demonstrating the binding of STAT3 on the *DAB2* promotor in P1 region. Data are expressed as the means ± standard deviations. **P < 0.01. **F** AGS cells with IL6 treatment and infection of *H pylori* strains (26,695 and 43,504). ChIP assay using STAT3 antibody was performed, followed by qPCR applying primers covering P1 region; ***P < 0.001. **G** Spearman’s correlation between *DAB2* and *IL6* was examined in the TCGA cohort

Next, to explore whether *H pylori* infection activated *DAB2* transcription, we transfected GC cells with *DAB2*-promoter reporter plasmids and followed by treatment of IL-6 or infection with *H pylori*. As shown, the findings illustrated that both IL-6 treatment and *H pylori* infection could enhance the *DAB2* promoter activity (Fig. 3A-B). We subsequently employed JASPAR (http://jaspar.genereg.net) to analyzed the *DAB2* promoter. The results illustrated 3 potential STAT3 binding sites on the *DAB2* promoter (Fig. 3C-D). To verify the STAT3 direct binding on the *DAB2* promoter, we employed chromatin immunoprecipitation (ChIP) assay followed by designing primers (P1–P3) that overlay the predicted binding sites (Fig. 3D). Notably, qRT-PCR assay showed amplification with P1 primers by CHIP, indicating direct binding of STAT3 on *DAB2* promoter (Fig. 3E). After GC cells treated with *H pylori* or IL-6, the ChIP assay illustrated a significant elevation in the P1 DNA fragments compared with control treatment (Fig. 3F). Furthermore, we confirmed the positive correlations between *IL-6* expression and the downstream target *DAB2* in TCGA cohort (Fig. 3G). Taken together, these findings indicated IL-6 and *H pylori* transcriptionally upregulated *DAB2* expression through direct binding of STAT3 on *DAB2* promoter, possibly through P1 binding sites.

### *H pylori* Infection Activates YAP1 Signaling via DAB2

**Fig. 4:**
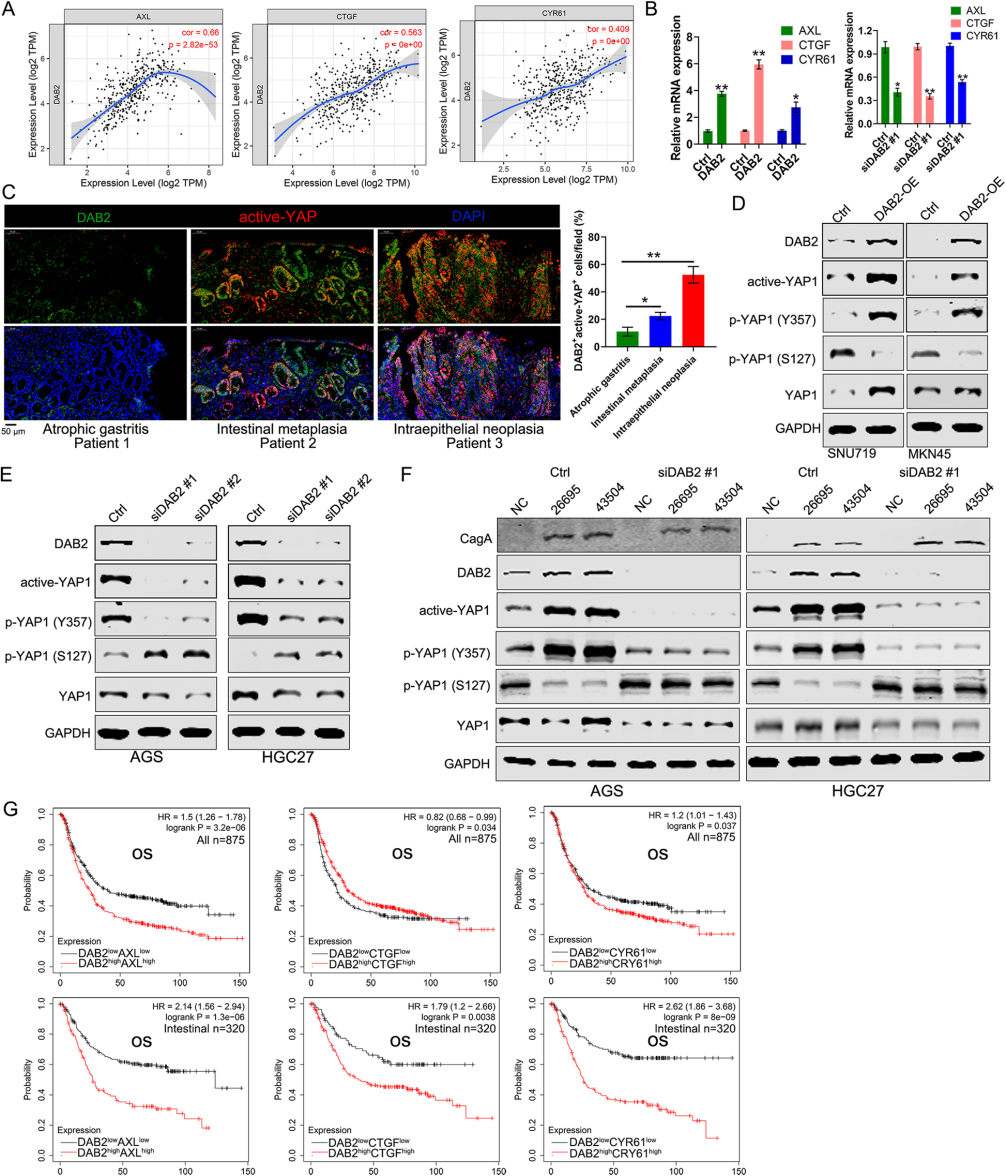
*H pylori* activated YAP1 signaling via *DAB2*. **A** Spearman’s correlation between *DAB2* and YAP1 signaling genes (*AXL*, *CTGF* and *CYR61*) was examined in the TCGA cohort. **B** The expressions of YAP1 signaling genes (*AXL*, *CTGF* and *CYR61*) were examined in AGS cells with *DAB2* overexpression or knockdown (*P < 0.05; **P < 0.01). **C** IF analysis shows the co-expression of DAB2 and active-YAP1 in patients with atrophic gastritis, intestinal metaplasia, and dysplasia (magnification ×200, scale bar, 50 μm). Quantification scores are shown on the right panel (*P < 0.05; **P < 0.01). **D-E** Western blot for DAB2, active-YAP1, p-YAP1 (S357), p-YAP1 (S127), and total YAP1 were performed in GC cells with *DAB2* overexpression or knockdown. **F** Western blot for DAB2, active-YAP1, p-YAP1 (S357), p-YAP1 (S127), and total YAP1 in AGS and HGC27 cell following *DAB2* silencing and *H pylori* infection. **G** Kaplan-Meier survival curve demonstrates an association between high expressions of *DAB2* and YAP1 signaling genes (*AXL*, *CTGF* and *CYR61*) and overall survival (OS) in overall GC patients and intestinal-type GC

Several reports have indicated that Hippo-YAP1 pathway was involved in *H pylori*-induced gastric carcinogenesis [16, 35]. Based on previous studies showing that YAP1 activation plays an important role in DAB2-regulated prometastatic activity of tumor-associated macrophages [26], we investigated whether *H pylori* activated YAP1 via DAB2. *AXL*, *CTGF*, and *CYR61* were regarded as YAP1 target genes, and were used to define “YAP1-on” status [36, 37]. Therefore, the correlations between *DAB2* and *AXL*/*CTGF*/*CYR61* were explored in TCGA dataset. The findings supported the positive correlations between *DAB2* and YAP1 downstream targets (*AXL*, *CTGF*, and *CYR61*) using Pearson’s correlation analysis (Fig. 4A). To validate the causal relationship between *DAB2* and “YAP1-on” status, we detected whether the activation of YAP1 depend on the expression of *DAB2* in AGS. *DAB2* silencing significantly decreased the mRNA levels of *AXL*, *CTGF*, and *CYR61*, whereas *DAB2* overexpression increased the mRNA levels of *AXL*, *CTGF*, and *CYR61* (Fig. 4B). Meanwhile, we detected the co-expression of DAB2 and active-YAP1 in patients with atrophic gastritis, intestinal metaplasia, and dysplasia. Our results indicated that both DAB2 and active-YAP1 expression increased with disease progression (Fig. 4C). Our fndings demonstrated that DAB2 overexpression activated YAP1 activity (upregulated p-YAP1^Y357^ and downregulated p-YAP1^S127^), whereas knockdown decreased YAP1 activity (Fig. 4D-E).

Then, we asked whether *H pylori* infection activated YAP1 signaling pathway in a DAB2-dependent manner. As expected, the findings showed that YAP1 activation mediated by *H pylori* infection was abolished by *DAB2* inhibition (Fig. 4F). In addition, by survival analysis of GC patients derived from TCGA dataset, combined survival analyses of *DAB2* and *AXL*, *CTGF*, or *CYR61* in GC patients demonstrated that high *DAB2*/*AXL*, *DAB2*/*CTGF*, and *DAB2*/*CYR61* levels are associated with worse OS. Furthermore, high *DAB2*/*AXL*, *DAB2*/*CTGF*, and *DAB2*/*CYR61* expression levels are more significantly associated with worse OS in GC patients with intestinal-type (Fig. 4G). Meanwhile, similar results were obtained with regard to DFS (Additional file 2: Fig. S4). Together, these data confirmed that *H pylori* infection activated YAP1 signaling via DAB2.

### DAB2 Stabilizes the ITGB3–SRC Complex and Enhances SRC Activity

**Fig. 5:**
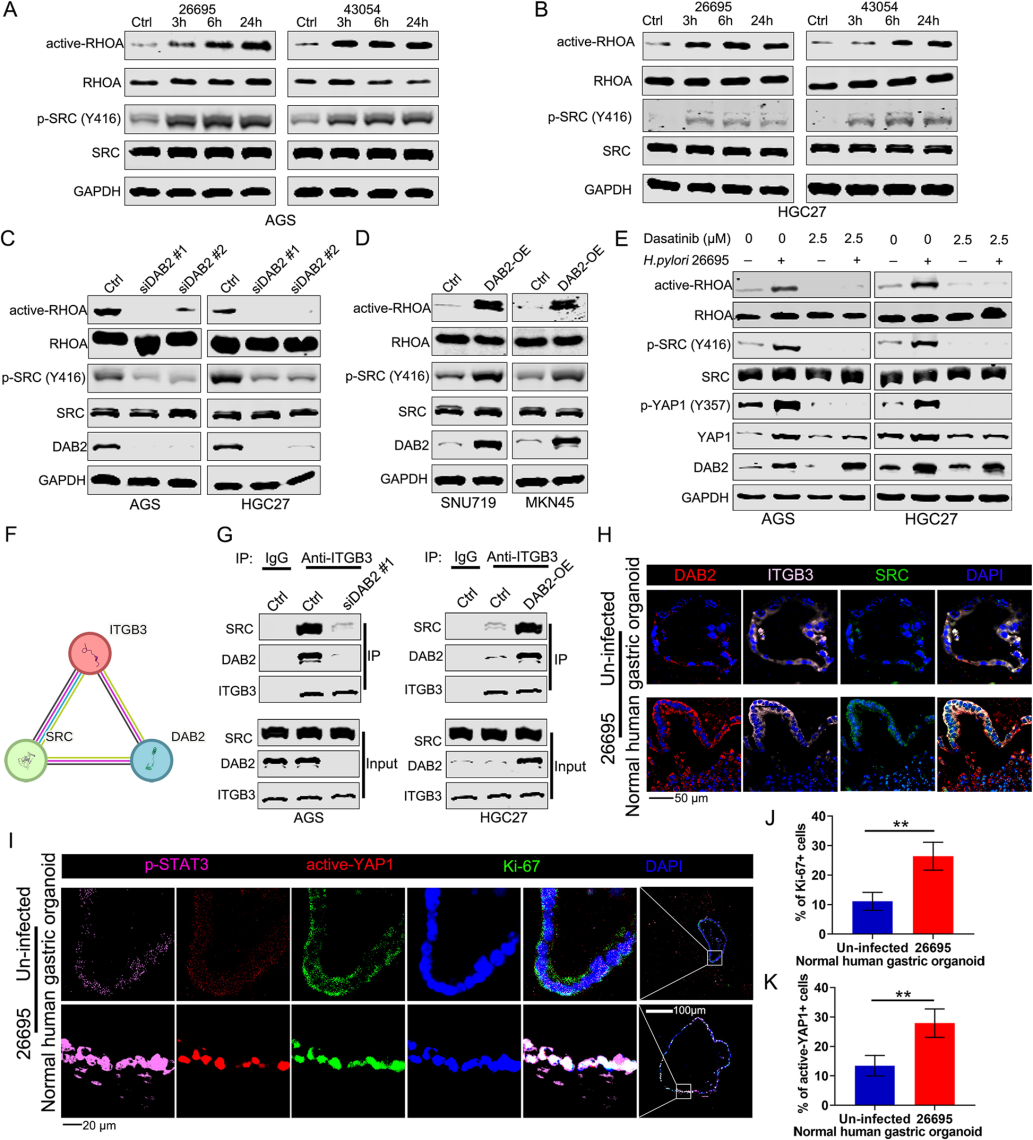
The DAB2 scaffolding function regulates YAP1 phosphorylation by promoting ITGB3-SRC interaction. **A-B** Western blot for active-RHOA, p-SRC (Y416), RHOA, and SRC were performed in GC cell lines treated with *H pylori* strains 26,695 and 43,504. **C-D** Western blot for active-RHOA, p-SRC (Y416), RHOA, and SRC were performed in gastric cancer cell lines with DAB2 overexpression or knockdown. **E** Western blot analysis for active-RHOA, p-SRC (Y416), RHOA, SRC, p-YAP1 (Y357), and YAP1 were performed in GC cell lines after treatment with SRC inhibitor dasatinib. **F** Physical interactions among DAB2, ITGB3, and SRC were predicted by STRING database (http://string-db.org/). **G** Co-immunoprecipitation of SRC and ITGB3 in AGS cells with or without *DAB2* knockdown (left) and in HGC27 cells with or without *DAB2* overexpression (right). **H** Representative IF images (magnification ×400, scale bars, 50 μm) of DAB2, ITGB3, and SRC in uninfected and *H pylori* 26,695–infected organoids derived from normal human gastric tissues; nuclei were stained with DAPI (blue). **I** Representative IF images (magnification ×400, scale bars, 20 μm) of P-STAT3, active-YAP1, and Ki67 in uninfected and *H pylori* 26,695–infected PDOs; nuclei were stained with DAPI (blue). **J-K** The quantification of nuclear Ki67 (**J**) and active-YAP1 (**K**) fluorescence is shown as the mean ± standard deviations of 3 independent fields; **P < 0.01

The tyrosine kinase SRC initiates the YAP1-dependent epiblast lineage differentiation through the direct phosphorylation of YAP1 [38]. In pathological conditions, such as GC and bacterial infection, *H pylori* have been shown to promote SRC kinase activity [33, 39]. Therefore, we postulated that SRC might mediate the activated effects of DAB2 on YAP1. To address the possibility, we treated AGS and HGC27 cells with *H pylori* and then monitored SRC activity. In line with our hypothesis, *H pylori* induced phosphorylation of the SRC activation (pY-416) [40], as well as src downstream effector-RHOA activation (Fig. 5A-B) [41]. To validate the causal relationship between DAB2 and SRC activation, we conducted *DAB2* knockdown or transient upregulation in GC cells. *DAB2* knockdown strikingly decreased the levels of SRC activation loop (pY-416) and active-RHOA, whereas *DAB2* overexpression reversed these effects (Fig. 5C-D). Moreover, inhibition of SRC activity by dasatinib could eliminate the effect of *H pylori* in promoting SRC activation (Fig. 5E).

Then, we tested the molecular mechanisms of DAB2 in promoting SRC phosphorylation. Integrins, including various combinations of integrin α and β subunits with different ECM ligand specificity, involving in YAP1 signaling pathway activation [42, 43]. Earlier studies have shown that DAB2 directly binds to integrin β1 (ITGB1), β3, and β5 to form DAB2–integrin complexes, which then regulates the communications between cell and ECM [44, 45]. On the basis of the findings that SRC interacted with ITGB3 leading to SRC activation [46, 47], we hypothesized that DAB2 might participate in the complex of ITGB3 with SRC to induce SRC phosphatase. To prove our hypothesis, we used STRING database (https://string-db.org/) to explore the protein-protein interactions, and the result showed that DAB2 could interact with ITGB3 and SRC by the analysis in STRING database (Fig. 5F), and this interaction was further determined by co-immunoprecipitation (Fig. 5G). IF staining demonstrated that DAB2, ITGB3 and SRC also colocalized in gastric organoids infected with *H pylori* (Fig. 5H). Furthermore, the IF staining on organoids from normal human gastric tissues illustrated a remarkable elevation in nuclear localization of Ki-67 (Fig. 5I-J) and a significant enhancement of active-YAP1 (Fig. 5I, K) after *H pylori* infection.

### AAV-mediated *DAB2* Downregulation Inhibits Tumour Progression in Organoid and PDX Model

**Fig. 6:**
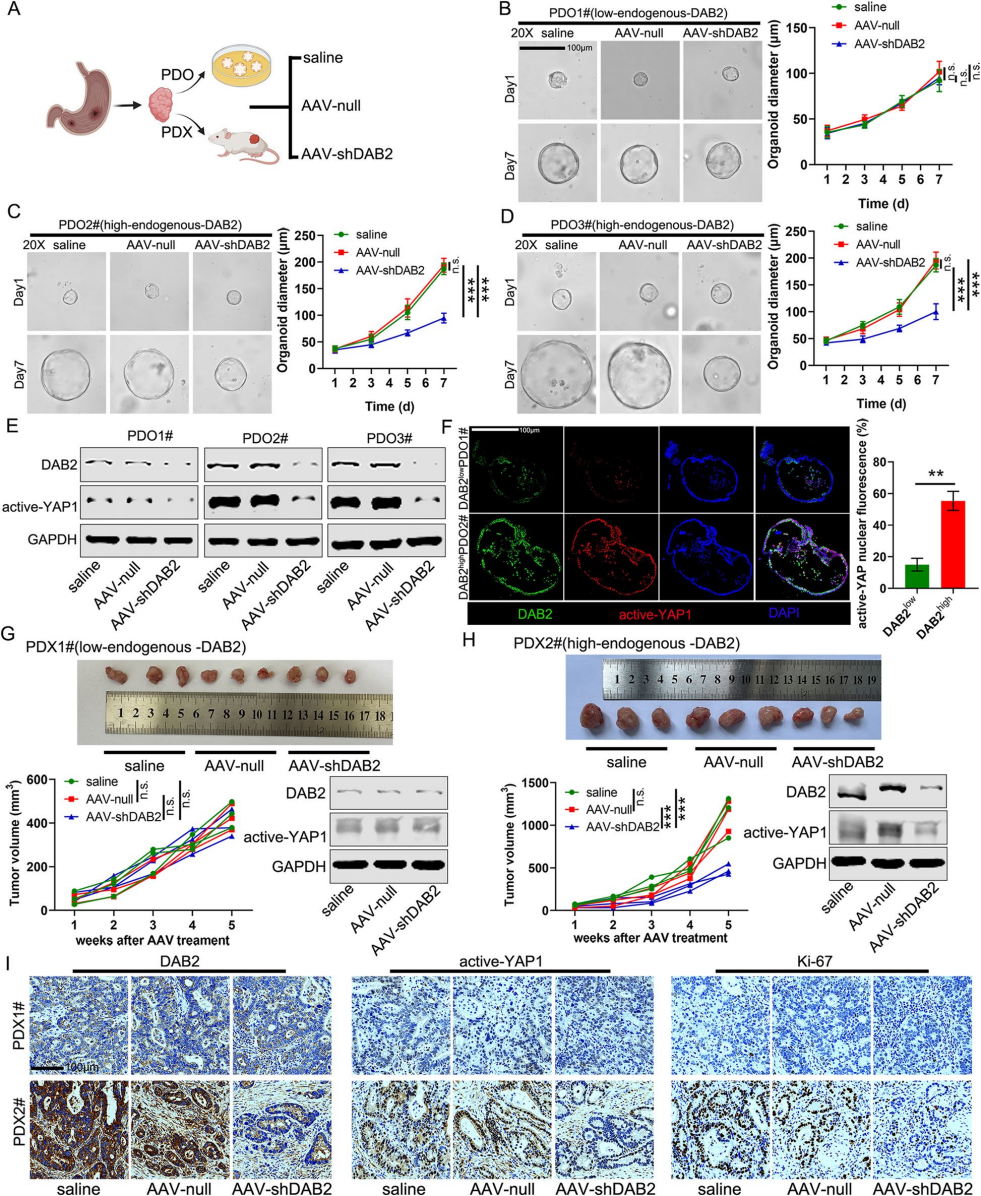
AAV-mediated *DAB2* knockdown inhibited tumour progression in human organoid model and PDX tumour mouse model. **A** Schematic diagram of the two mouse models: patient-derived organoids (PDO) model and patient-derived xenografts (PDX) model. **B-D** Parental PDO1#/2#/3# were digested into single cell suspensions and then seeded into 24-well plates. (The endogenous *DAB2* expression is low in PDO1# but high in PDO2#/3#) Saline, AAV-null and AAV-sh*DAB2* were added into each group. Representative images of organoids in each group at day 1 (baseline) and day 7 (after treatment) were shown. The diamater of organoids was measured (***P < 0.001). **E** The expression of DAB2 and active-YAP1 in PDOs were analyzed by western blot after receiving the treatment of saline, AAV-null and AAV-sh*DAB2*. **F** The expression of DAB2 and active-YAP1 in PDOs were analyzed by IF in DAB2^low^ PDO1# and DAB2^high^ PDO2#. Quantification of positive active-YAP1 cells was shown; **P < 0.01 (right panel). **G-H** PDX models from the patient1#/2# were randomized into three groups and then administrated with saline, AAV-null and AAV-sh*DAB2*. The tumors were harvested on day 35. The expression of DAB2 and active-YAP1 in PDOs were analyzed by western blot after receiving the above treatment. The volume of PDXs was also measured (***P < 0.001). I) The expression of DAB2, active-YAP1, and Ki-67 were analyzed by IHC using paraffin-embedded tumor tissues from PDX mouse model

To explore whether DAB2 could be used as a therapeutic target in GC, an AAV- mediated shRNA was constructed, and patient-derived organoid (PDO) model and patient-derived xenografts (PDX) mouse model were adopted (Fig. 6A). Organoids from 3 GC tumor tissues were used in the experiment. Patient 1# and 2# showed high *DAB2* expression levels, while Patient 3# showed a low DAB2 level. These organoids were subsequently treated with AAV-sh*DAB2*, AAV-null, and saline. In *DAB2*-low organoids, the growth of PDOs in AAV-sh*DAB2* group showed no significant difference compared with the control group after 7 days of treatment (Fig. 6B). However, the growth of PDOs was significantly reduced by AAV-sh*DAB2* in *DAB2*-high organoids (Fig. 6C-D). Furthermore, our Western blot and IF results confirmed that knockdown of *DAB2* expression significantly inhibited YAP1 activation (Fig. 6E), and the level of YAP1 activation in DAB2^high^ GC patients was higher that in DAB2^low^ GC patients (Fig. 6F). Furthermore, AAV-sh*DAB2* treatment was performed in PDX mouse models. *DAB2* expression in PDX1# was lower than that in PDX2#. In *DAB2* low expression PDX-1#, no therapeutic effect of AAV-sh*DAB2* and no difference of YAP1 activation were observed (Fig. 6G). However, the tumour growth and YAP1 activation were significantly inhibited by AAV-sh*DAB2* in *DAB2* high expression PDX2# (Fig. 6H). Furthermore, the IHC staining also showed a significant decrease of DAB2, Ki-67 and active-YAP1 nuclear localization by AAV-sh*DAB2* in *DAB2*-high PDX tumour, whereas there was no significant difference in *DAB2*-low PDX tumour (Fig. 6I).

### *DAB2* Depletion Inhibited Expansion of Gastric Cancer Cells via YAP1

**Fig. 7:**
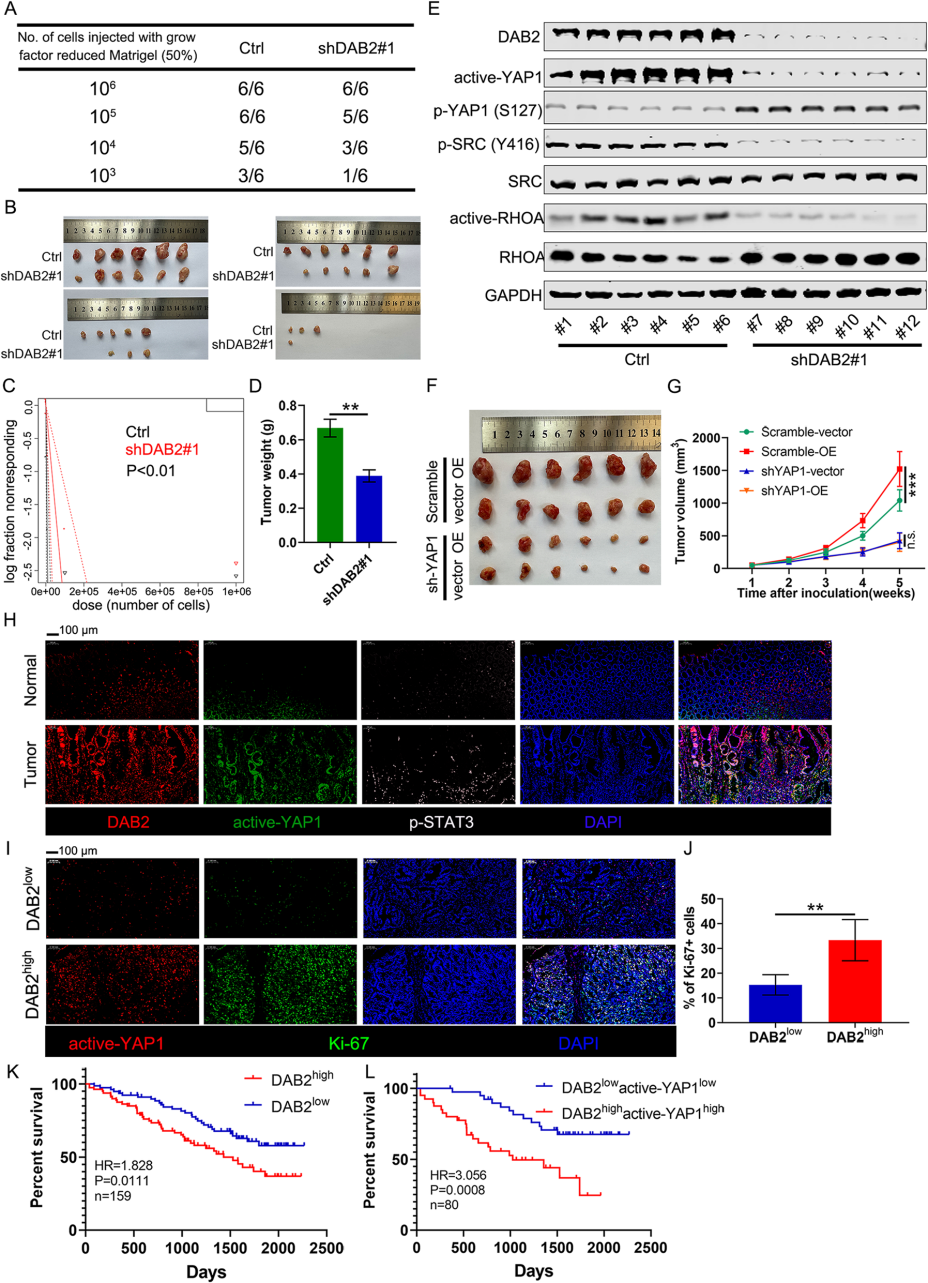
*DAB2* regulates gastric tumorigenesis in a YAP1-dependent manner. **A-C** HGC27 cells with/without *DAB2* knockdown were serially diluted and xenografted into nude mice subcutaneously. (**A-B**) The tumor cell numbers injected and frequency of tumor formation at day 35. (**C**) The probability estimates calculated with Extreme Limiting Dilution Analysis (ELDA) software (http://bioinf.wehi.edu.au/software/elda/). A significant difference in tumor formation capacity was observed between the control and sh*DAB2* groups. **D** Tumor weight for subcutaneous tumor xenografts with shRNA knockdown or control (1 million cells dose group); **P < 0.01. **E** Western blot for DAB2 and its downstream signaling genes for xenograft tumors with/without *DAB2* silencing (from 1 million cells dose group). **F-G** Tumour growth curves of Scramble-vector, Scramble-OE, sh*YAP1*-vector, and sh*YAP1*-OE groups were shown. ***P < 0.001. **H** Representative IF images (magnification ×100, scale bars, 100 μm) of DAB2, active-YAP1, and p-STAT3 in human adjacent normal tissue and tumor; nuclei were stained with DAPI (blue). **I** Representative IF images (magnification×100, scale bars, 100 μm) of active-YAP1 and Ki-67 in human DAB2^high^ and DAB2^low^ GC tissues; nuclei were stained with DAPI (blue). **J** The quantification of nuclear Ki67 fluorescence is shown as the mean ± standard deviations of DAB2^high^ and DAB2^low^ GC tissues; **P < 0.01. **K-L** Kaplan-Meier survival curve indicates that GC patients with high expression of DAB2 (**K**) and DAB2 + active-YAP1 (**L**) have poor overall survival (OS) compared with GC patients with low expression. HR, hazard ratio

To further investigate the in vivo tumourigenic ability of *DAB2*, limiting dilution assays showed significantly lower tumor incidence and tumor weight in sh*DAB2* HGC27 cell lines (Fig. 7A-D). Western blots confirmed downregulation of SRC/YAP1 and SRC/RHOA signaling axis in the *DAB2* downregulation cells, and validated tumor growth inhibition of *DAB2* (Fig. 7E). Additionally, to examine whether DAB2 enhanced GC expansion via YAP1 activation, we next performed blocking assays in vivo. DAB2 did not induce GC cell proliferation when YAP1 was downregulated (Fig. 7F-G). Taken together, these observations showed that *DAB2* overexpression or deletion in GC cells affect cell proliferation in a YAP1-dependent manner.

We subsequently determined DAB2 protein expression via IF staining in a TMA with 77 evaluable GC cases and adjacent nontumor tissues. Representative DAB2, active-YAP1, and p-STAT3 positive and negative images were shown in Fig. 7H. In addition, IF staining of active-YAP1 and Ki-67 was performed in another TMA containing 149 evaluable GC cases. The expression of Ki-67 in DAB2^high^ expression group was higher than that in the DAB2^low^ expression group (Fig. 7I-J), suggesting that DAB2 may be involved in cell proliferation in gastric carcinogenesis. Kaplan-Meier survival analysis showed that DAB2^high^ patients had a less overall survival compared with DAB2^low^ patients (Fig. 7K). Furthermore, high DAB2/active-YAP1 expression levels are more significantly associated with worse overall survival in GC patients (Fig. 7L). Collectively, these findings confirmed the function of YAP1 in DAB2 mediated protumor effects in GC.

## Discussion

The identification and characterisation of specific gene expression signatures and *H pylori* infection-driven genes in the process of GC development provides a better understanding for the molecular mechanisms of gastric carcinogenesis. In the present study, we used a range of sequencing data to search the crucial *H pylori* infection-driven oncogenes, and *DAB2*-mediated malignant phenotypes were revealed. In particular, our results showed the direct transcriptional upregulation of *DAB2* via STAT3 in response to *H pylori* infection. Here our data demonstrated the function of DAB2 in promoting the proliferation of GC cells through activating SRC/YAP1 signaling axis, and the activation of SRC kinase is the required downstream of DAB2 to unleash the YAP1-mediated gastric tumorigenesis.

The STAT3 signaling pathway is activated in different cancers, including GC [48–50]. The dysregulation of Hippo signaling pathway and YAP/TAZ-TEAD activity is involved in cancer biology, cancer stem cell renewal, cancer immunity, and tumorigenesis [51–53]. Nevertheless, the interaction effect of STAT3 and Hippo pathways are not well elucidated in gastric tumorigenesis, especially in *H pylori*-driven gastric carcinogenesis. In our current study, the findings demonstrated that STAT3 and Hippo signaling pathways exerted key roles in *H pylori* infection-induced gastric tumorigenesis. These findings further illustrated that *H pylori*-mediated STAT3 activation and activated the transcription of *DAB2*, which further activated YAP1 and upregulated the transcription of the downstream target genes, and therefore promotes gastric carcinogenesis.

**Fig. 8:**
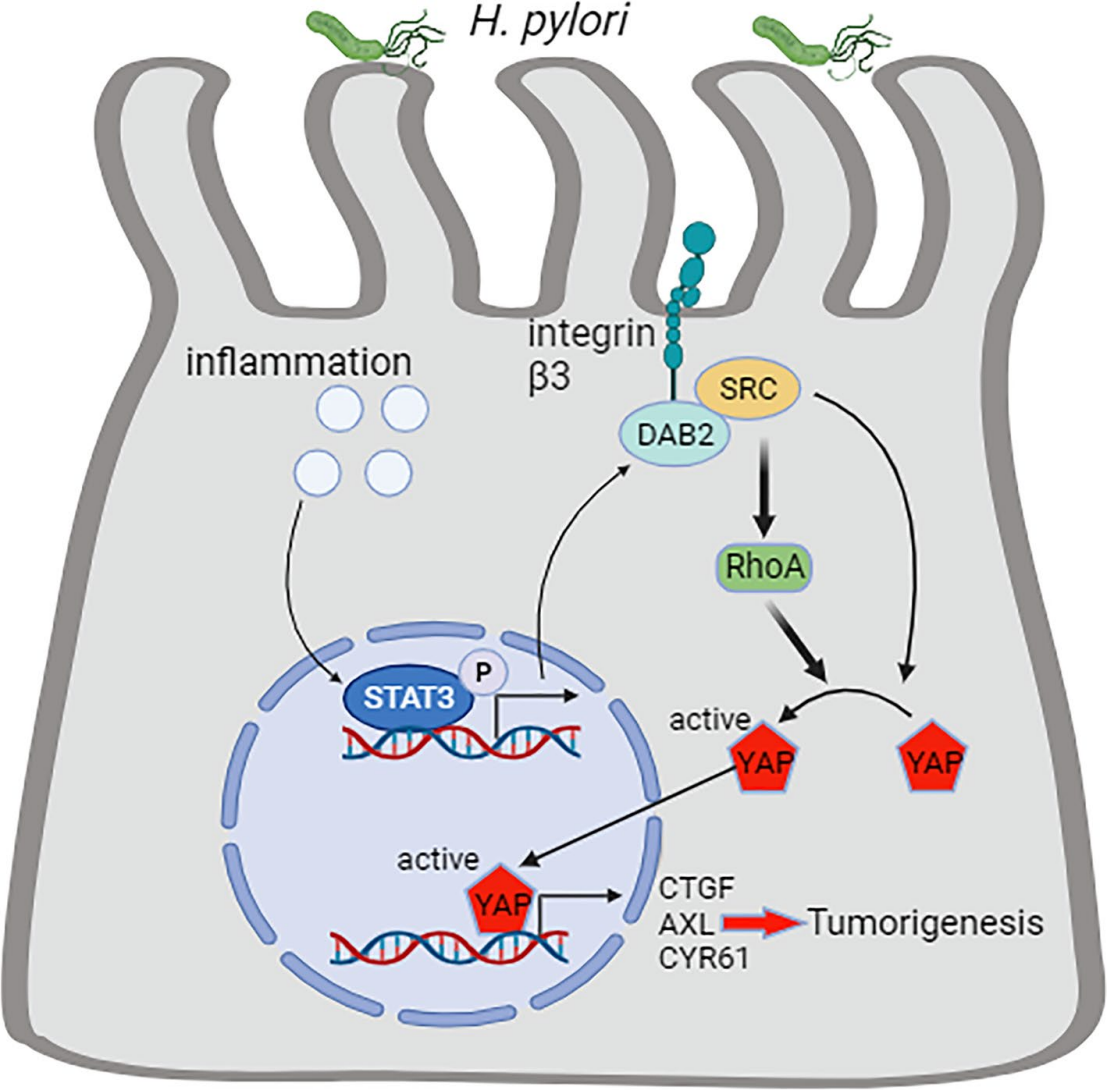
Schematic model for *DAB2*-mediated YAP regulation via a non-canonical signalling complex in *H pylori*-driven gastric tumorigenesis. DAB2 served as a scaffold protein to form a complex with integrin beta 3 (ITGB3) and SRC proto-oncogene non-receptor tyrosine kinase, facilitating the YAP1 transcriptional activity in response to *H pylori* infection

*DAB2* was originally identified as a tumor suppressor gene in 1994 [21]. However, in the present study, the results indicated the oncogenic characteristics of DAB2 in GC. Furthermore, previous studies showed contradictory results indicating a pro-tumor effect of DAB2. TGFβ through DAB2 activated the FAK which subsequently promoted the activation of ITGB1, enhancing EMT and preventing cell apoptosis [25]. Our study reveals, for the first time, DAB2’s oncogenic function depended on the SRC-dependent activation of YAP1. Mechanistically, DAB2 could function as a scaffold protein to enhance the interaction between ITGB3 and SRC, leading to elevated SRC phosphorylation, and thus promoting YAP1-dependent translation. DAB2 functions as a multimodular scaffold protein by interacting with other proteins [54]. A previous research reported that the clathrin adaptor DAB2 recruited EH domain scaffold proteins to regulate ITGB1 endocytosis [55]. DAB2 has been identified as the crucial adaptor protein binding PP2A to apoER2 to form an apoER2-DAB2-SHC1 complex involved in thrombosis [56]. These findings, together with our results, imply that DAB2 acted as a scaffold protein to promote protein-protein interaction. ITGB3 could promote SRC activity by directly interacting with SRC in the cell membrane [57]. However, in the current study, we uncovered a previously unknown mechanism that DAB2 promoted SRC activity by directly interacting with SRC and ITGB3 to enhance the protein interactions (Fig. 8).

*H pylori* was identified as a class I carcinogen and recognized as the most important risk factor for GC over the past few decades [1], and *H pylori*-related gastric tumorigenesis become the focus of current research. However, the molecular mechanisms of *H pylori* infection-mediated gastric carcinogenesis remains incompletely understood. Recent study demonstrated that *H pylori* infection enhanced NF-κB/RASAL2/PP2A/AKT/β-catenin axis activation to promote gastric tumorigenesis [58]. Furthermore, upregulation of FGFR4 by *H pylori* infection through STAT3 to form a forward activation feedback loop, participating in gastric tumorigenesis [33]. In particular, *H pylori* induced the pro- tumorigenic STAT3 signaling pathway to promote colorectal carcinogenesis [34]. Although recent studies have recognized the effect of *H pylori* in gastric and colorectal tumorigenesis, the intricate molecular players and mechanisms remain elusive. The current study showed a previously unknown molecular mechanism of DAB2 in the activation of SRC/YAP1 signaling to promote *H pylori* induced gastric carcinogenesis. Our data illustrated that DAB2 acted as a crucial mediator in *H pylori*-induced GC via a YAP1–dependent manner, and we further confirmed a cascade from STAT3 to YAP1 signaling. Additionally, coincident DAB2 and active-YAP1 upregulation was observed in GC tumor tissues and indicated a poor prognosis, indicating that elevated DAB2 and active-YAP1 expression levels served as predictive biomarkers for GC. More importantly, we confirmed these findings in different pathological stages of Correa’s cascade, supporting the novel role of DAB2 in *H pylori*-mediated gastric carcinogenesis.

In conclusion, this study demonstrated DAB2 exerts a crucial role in *H pylori* infection-mediated gastric tumorigenesis. Our findings suggest that the STAT3/DAB2/SRC/YAP1 signaling axis enhances the tumorigenic cell properties, contributing to the development of new therapeutic strategies. Further identification of the YAP1-regulated genes in gastric tumorigenesis would be necessary in the future.

### Supplementary Information

**Supplementary Material 1.**

## Data Availability

The datasets supporting the conclusions of this article are included within the article (and its Additional files) and available from the corresponding author on reasonable request.

## Abbreviations

*H pylori*: *Helicobacter pylori*
GC: Gastric cancer
*DAB2*: Disabled-2
*STAT3*: Signal transducer and activator of transcription 3
*YAP1*: Yes-associated protein 1
*ITGB3*: Integrin beta 3
*SRC*: SRC proto-oncogene non-receptor tyrosine kinase
*RHOA*: Ras homolog family member A
*NF-κB*: Nuclear factor κB
*JNK*: c-Jun NH2-terminal kinase
*MAPK*: Mitogen-activated protein kinase
*TGFβ*: Transforming growth factor beta
ECM: Extracellular matrix
EMT: Epithelial to mesenchymal transition
*FAK*: Focal adhesion kinase
TAMs: Tumor-associated macrophages
TMA: Tissue microarray
GEO: Gene Expression Omnibus
TCGA: The Cancer Genome Atlas
*AXL*: AXL receptor tyrosine kinase
*CTGF*/*CCN2*: Cellular communication network factor 2
*CYR61*/*CCN1*: Cellular communication network factor 1
DAPI: 4, 6-diamidino-2-phenylindole
IF: Immunofluorescence
AAV: Adeno-Associated Virus
DEGs: Differential expression genes
OS: Overall survival
GSEA: Gene set enrichment analysis
ChIP: Chromatin immunoprecipitation
PDO: Patient-derived organoid
PDX: Patient-derived xenografts
