# Supplementary material for: STAT3–mediated up-regulation of DAB2 via SRC-YAP1 signaling axis promotes Helicobacter pylori-driven gastric tumorigenesis
Source: Biomark Res. 2024 Mar 13;12:33. doi: 10.1186/s40364-024-00577-x (PMC10935867; doi:10.1186/s40364-024-00577-x)
Supplement: Supplementary file 1 — Supplementary Material 1. [file 40364_2024_577_MOESM1_ESM.docx]

**Additional file**

**STAT3–Mediated Up-Regulation of DAB2 via SRC-YAP1 Signaling Axis Promotes Helicobacter Pylori-Driven Gastric Tumorigenesis**

Additional file 1: Table S1. Antibody List

| Antibody | Manufacturer | Catalogue number | Application | Working dilution |
| --- | --- | --- | --- | --- |
| DAB2 | Santa Cruz | sc-136964 | IHC, IF | 1:100, 1:50 |
| Ki-67 | abcam | ab15580 | IHC | 1:100 |
| CagA | Santa Cruz | sc-28368 | WB | 1:1000 |
| DAB2 | abcam | ab256524 | WB | 1:1000 |
| p-STAT3 (Y705) | Cell Signaling | #73533 | WB | 1:1000 |
| STAT3 | Cell Signaling | #9139 | WB | 1:1000 |
| GAPDH | proteintech | 60004-1-Ig | WB | 1:5000 |
| active-YAP1 | abcam | ab205270 | IHC, IF, WB | 1:100, 1:50, 1:1000 |
| p-YAP1 (Y357) | abcam | ab62751 | IHC, IF, WB | 1:100, 1:50, 1:1000 |
| p-YAP1 (S127) | Cell Signaling | #13008 | WB | 1:1000 |
| YAP1 | Cell Signaling | #14074 | WB | 1:1000 |
| RhoA | Cell Signaling | #2117 | WB | 1:1000 |
| p-SRC (Y416) | Cell Signaling | #59548 | WB | 1:1000 |
| SRC | Cell Signaling | #2109 | WB | 1:1000 |
| ITGB3 | Santa Cruz | sc-46655 | WB | 1:1000 |
| IRDye® 800cw Goat Anti-Mouse IgG | Li-COR | #926-32210 | WB | 1:10000 |
| IRDye® 800cw Goat Anti-Rabbit IgG | Li-COR | #926-32211 | WB | 1:10000 |

Additional file 1: Table S2. Sequences of Primers

| Gene | Oligo name | Oligo sequence |
| --- | --- | --- |
| P1  P2  P3  *DAB2*  *AXL*  *CTGF*  *CYR61*  *GAPDH* | Forward primer  Reverse primer  Forward primer  Reverse primer  Forward primer  Reverse primer  Forward primer  Reverse primer  Forward primer  Reverse primer  Forward primer  Reverse primer  Forward primer  Reverse primer  Forward primer  Reverse primer | *ACTACGTGCTGCCTCTGACT*  *TCCTAATGAGTGTTAAC*  *CTGCGGAGTACCTTCTACCG*  *TCTGATACAATCAGAGATTC*  *GAGACACTAATTTCTTCTAAG*  *GAGCTTCGTTTCACAGATCCG*  *TGGTACTGAGGTTTGCTGCC*  *CTGCCGCCATTCCCTTTAGT*  *GTATCAAGGCCAGGACACCC*  *AAGGTTCCTTCACTGGGCG*  *ATGGTGCTCCCTGCATCTTC*  *TCTTCCAGTCGGTAAGCCGC*  *AGCGTTTCCCTTCTACAGGC*  *TTCTCCAATCGTGGCTGCAT*  *GTCTCCTCTGACTTCAACAGCG*  *ACCACCCTGTTGCTGTAGCCAA* |
| *DAB2* siRNA #1 | 5’-*CCUGCCAGUUACCAAAUCUTT*-3’ | |
| *DAB2* siRNA #2 | 5’-*CCAGCAGUGAGAACUCAAATT*-3’ | |
| *DAB2* shRNA#1 | *AGCCAAGACTCTATGATGAAA* | |
| *DAB2* shRNA#2 | *TTGCTTGCATCAGACATCTTT* | |
| *ITGB3* siRNA | 5’-*UGUUUGUAGCCAAACAUGGGC*-3’ | |

Additional file 1: Table S3. Detailed clinical information of GC patients for human organoids model.

| Patient ID | Age | Gender | TNM  stage | Pathological  stage | GC differentiation grade |
| --- | --- | --- | --- | --- | --- |
| Organoid1# | 56 | Female | T3N1M0 | IIB | poorly |
| Organoid2# | 65 | Male | T2N1M0 | IIA | poorly |
| Organoid3# | 68 | Male | T2N0M0 | IB | moderately |

Additional file 1: Table S4. Detailed clinical information of GC patients for PDX model.

| Patient ID | Age | Gender | TNM  stage | Pathological  stage | GC differentiation grade |
| --- | --- | --- | --- | --- | --- |
| PDX1# | 63 | Male | T3N1M0 | IIB | poorly |
| PDX2# | 68 | Male | T2N1M0 | IIA | poorly |

Additional file 2: Figure S1.


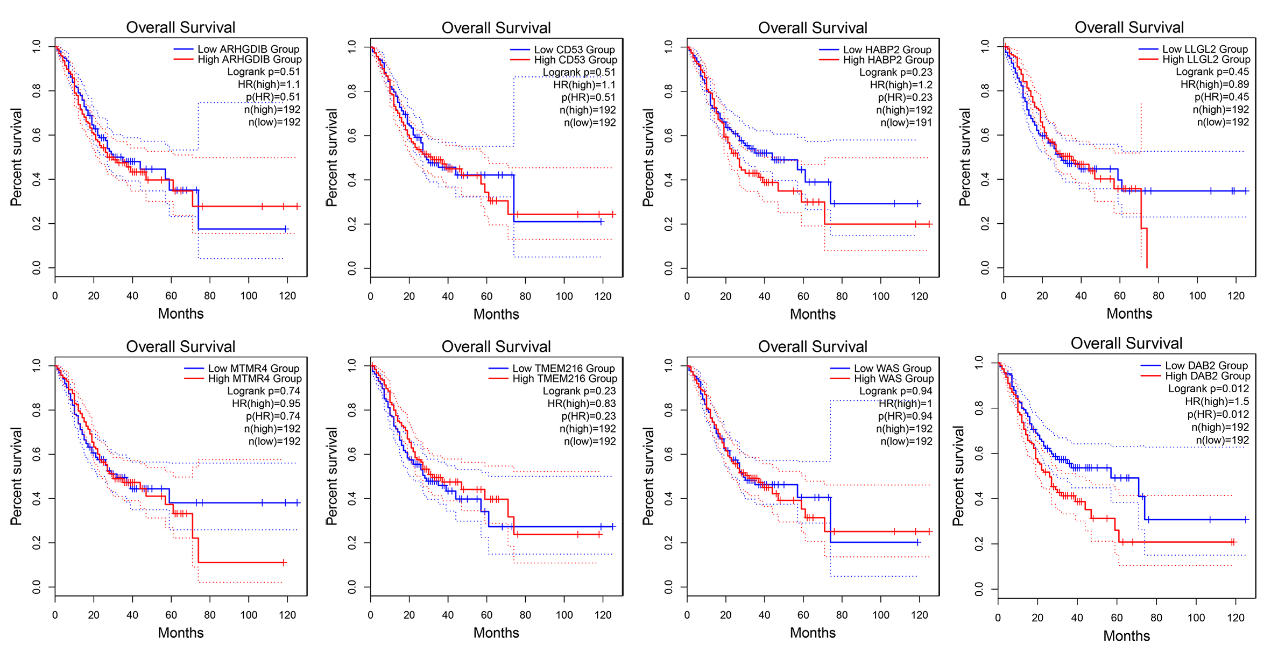


Additional file 2: Fig. S1. The survival analysis of the 8 DEGs in TCGA cohort.

Additional file 2: Figure S2.


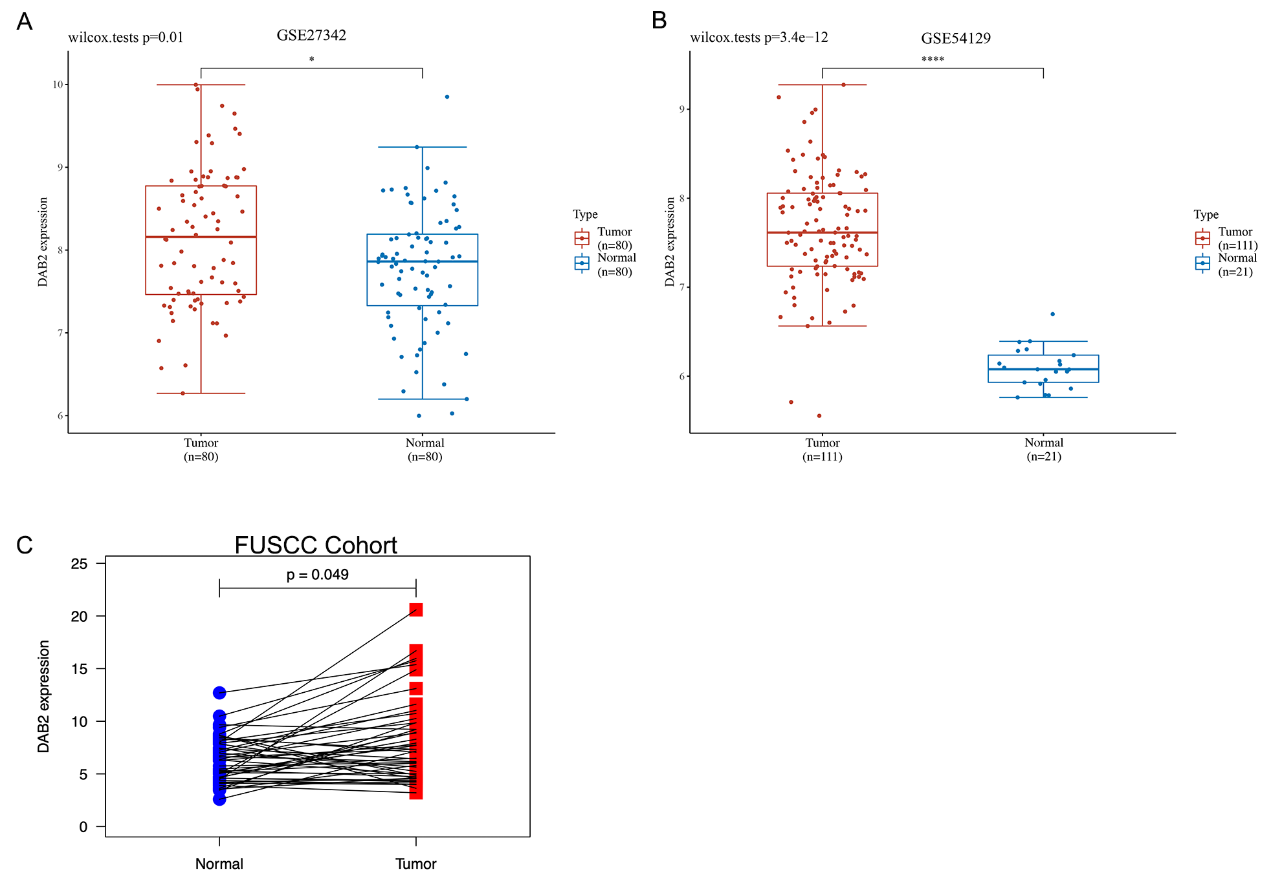


Additional file 2: Fig. S2. (A) The relative expression of DAB2 in 80 paired GC tissues from GSE27342; **P* < 0.05. (B) The relative expression of DAB2 in unpaired 111 GC tissues and 21 adjacent normal tissues from GSE54129; *****P* < 0.0001. (C) The relative expression of DAB2 in 50 paired GC tissues from our FUSCC cohort.

Additional file 2: Figure S3.


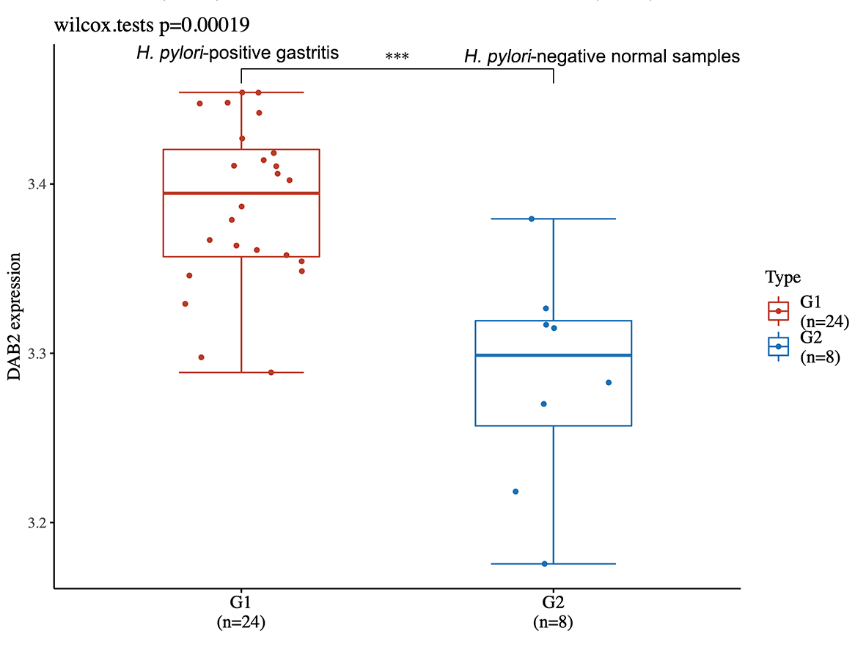


Additional file 2: Fig. S3. The relative expression of *DAB2* in 24 human *H pylori*-positive gastritis and 8 *H pylori*-negative normal samples from GSE60427; ****P* < 0.001.

Additional file 2: Fig. S4.


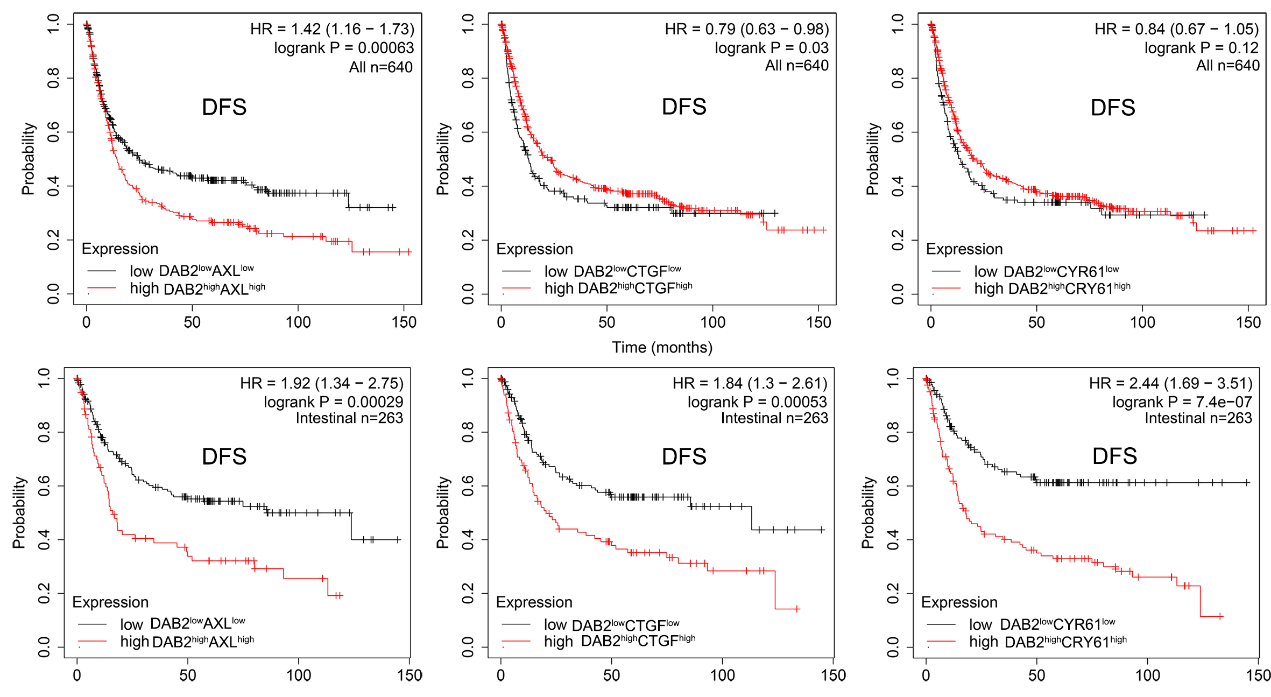


Additional file 2: Fig. S4. Kaplan-Meier survival curve demonstrates an association between high expressions of *DAB2* and YAP1 signaling genes (*AXL*, *CTGF* and *CYR61*) and disease-free survival (DFS) in overall GC patients and intestinal-type GC.
